# Disease Classification and Biomarker Discovery Using ECG Data

**DOI:** 10.1155/2015/680381

**Published:** 2015-11-24

**Authors:** Rong Huang, Yingchun Zhou

**Affiliations:** Department of Statistics and Actuarial Sciences, East China Normal University, Shanghai 200241, China

## Abstract

In the recent decade, disease classification and biomarker discovery have become increasingly important in modern biological and medical research. ECGs are comparatively low-cost and noninvasive in screening and diagnosing heart diseases. With the development of personal ECG monitors, large amounts of ECGs are recorded and stored; therefore, fast and efficient algorithms are called for to analyze the data and make diagnosis. In this paper, an efficient and easy-to-interpret procedure of cardiac disease classification is developed through novel feature extraction methods and comparison of classifiers. Motivated by the observation that the distributions of various measures on ECGs of the diseased group are often skewed, heavy-tailed, or multimodal, we characterize the distributions by sample quantiles which outperform sample means. Three classifiers are compared in application both to all features and to dimension-reduced features by PCA: stepwise discriminant analysis (SDA), SVM, and LASSO logistic regression. It is found that SDA applied to dimension-reduced features by PCA is the most stable and effective procedure, with sensitivity, specificity, and accuracy being 89.68%, 84.62%, and 88.52%, respectively.

## 1. Introduction

In the recent decade, classification and feature discovery have attracted more and more attention in many areas of sciences, such as biology, medicine, chemistry, and economics. In particular, disease classification and biomarker discovery become increasingly important in modern biological and medical research. ECGs are comparatively low-cost and noninvasive in screening and diagnosing heart diseases. With the development of personal ECG monitors, large amounts of ECGs are recorded and stored; therefore, fast and efficient algorithms are called for to analyze the data and make diagnosis. In this paper, an efficient and easy-to-interpret procedure of cardiac disease classification is developed through novel feature extraction methods and comparison of classifiers. Such procedure can be applied to other similar classification and biomarker identification problems.

Classification of ECGs usually consists of three steps: signal preprocessing, feature extraction, and classification. Features that have been used in characterizing the ECGs include heartbeat interval features, frequency-based features, higher order cumulant features, Karhunen-Loeve expansion of ECG morphology, and hermite polynomials [1–5]. Previous methods of ECG classification include linear discriminants [6], decision tree [7–9], neural networks [1, 10, 11], support vector machine [2–5], and Gaussian mixture model algorithm [12]. Some researchers perform disease detection using ECG data along with other clinical measurements [8, 10]. However, for those methods which used coefficients of various basis functions as features for classification, such as the wavelet coefficients, the coefficients are usually not easy to interpret clinically. And for those methods which only chose certain parts on ECGs for classification, their selection might be subjective and might cause bias in the final results. A simple method using 12-lead ECG data is developed in [13], which measures eight temporal intervals for each of the 12 leads, and uses the number of the intervals exceeding the control value by two standard deviations as a disease indicator. Although the sensitivity and specificity of this method are relatively high compared to other methods (72% and 92%, resp.), it does not include variables other than temporal measurements and cannot capture the features well when the distributions of the measurements are heavy-tailed or skewed or exhibit other nonnormal patterns.

In this paper, we use novel methods to extract interpretable features and compare the performance of different types of classifiers. The novelties of this paper are threefold. Firstly, we extract features by taking quantiles of the distributions of measures on ECGs, while commonly used characterizing feature is the mean. This is motivated by our observation that the distributions of the measures of the diseased group are often skewed, heavy-tailed, or multimodal, whose features cannot be well captured by the mean. It turns out that the performance of quantile measures is better than that of the mean measures. Secondly, we include commonly used measurement variables on ECGs without preselection and use dimension reduction methods to identify biomarkers. Our method is useful when the number of input variables is large and no prior information is available on which ones are more important. Thirdly, we compare the performance of three frequently used classifiers applied both to all features and to dimension-reduced features by PCA. The three methods are from classical to modern: stepwise discriminant analysis (SDA), SVM, and LASSO logistic regression. It is found that SDA on dimension-reduced features by PCA is the most stable and effective procedure, with sensitivity, specificity, and accuracy being 89.68%, 84.62%, and 88.52%, respectively.

## 2. Data Description and Signal Preprocessing

The real data used in the paper is PTB data set available at http://www.physionet.org/physiobank/database/ptbdb/. It contains ECG records of 290 volunteers; some are healthy and others diagnosed with certain cardiac diseases. Each subject has several 1-2-minute-long records of standard 12-lead ECGs, accompanied with his/her gender, age, and clinical diagnosis results. There are 219 male and 81 female subjects, age ranging from 17 years to 87 years with an average age of 57.2. Among the 290 subjects, 44 subjects have missing information in their records, so 246 subjects with 498 ECG records are used in classification. The data contains five health status categories: healthy, myocardial infarction, cardiomyopathy, atrioventricular bundle branch block, and rhythm disorders. Since the sample sizes for cardiomyopathy, atrioventricular bundle branch block, and rhythm disorders are too small to generate a reasonable classifier, we put the disease groups together to form a “Disease” category; see Table 1. The sampling frequency of the data set is 1000 Hz, and the precision is 16 bits. The input voltage is about 16 mV, and the compensation offset voltage is about 300 mV.

We use single-lead data (MLII) for classification, noting that the methods can be applied to 12-lead data as well. The ECGPUWAVE function in the WFDB package available at http://www.physionet.org/physiotools/ecgpuwave/ is applied to mark the start, peak, and end points of the P wave, the QRS complex, and the T wave. This function also provides the T wave type of each heartbeat which is one of the features used in classification.

## 3. Feature Extraction

ECG measurements for each heartbeat are obtained based on the annotations by the ECGPUWAVE function. Four types of features are considered as input variables for classification: T wave type, time span measurements, amplitude measurements, and the slopes of waveforms. Below are detailed descriptions about these features.

### 3.1. Four Types of Features

*(1) T Wave Type.* The ECGPUWAVE function labels 6 types of T waves for each beat: Normal, Inverted, Positive Monophasic, Negative Monophasic, Biphasic Negative-Positive, and Biphasic Positive-Negative based on the T wave morphology. This is the only categorical variable considered.

*(2) Time Span Measurements.* Six commonly used time span measurements are considered: the length of the RR interval, PR interval, QT interval, P wave, QRS wave, and T wave.

*(3) Amplitude Measurements.* The amplitudes of P wave, R-peak, and T wave are used as input variables. To measure the P wave amplitude, we first estimate the baseline by taking the mean of the values in the PR segment, ST segment, and TP segment (from the end of the T wave to the start of the P wave of the next heartbeat), then subtract the maximum and minimum values of the P wave by the estimated baseline, and take the one with a bigger absolute value as the amplitude of P wave. Other amplitude measurements are obtained similarly.

*(4) The Slopes of Waveforms.* The slopes of waveforms are also considered to measure the dynamic features of a heartbeat. Each heartbeat is split into nine segments and the slope of the waveform in each segment is estimated by simple linear regression.  Table 2 lists the nine waveforms with definitions.

### 3.2. Adjustment for Time Span Measurements

It is well documented that the QT interval is related to the RR interval and needs to be adjusted to be compared among beats. Similarly, other time span measures also tend to change with the RR interval. Note that a commonly used clinical correction for QT interval is Bazett's formula [14]: ${QT}_{c}=QT/\sqrt{RR}$, where QT_*c*_ represents the adjusted value of QT interval. We thus apply Model (1) to the data of healthy subjects to find correction formulas for the other time span measurement variables:

(1) $$\begin{array}{c} y=\beta {RR}^{\alpha }+\varepsilon , \end{array}$$

where *y* represents a time span measurement variable and *ε* is an error term. Through investigating the scatterplots between *y* and RR, the range of *α* in (1) for all these measurement variables is roughly within [0,1]. Though *α* is a continuous variable, we discretize its range and select a best value of *α* among {0, 0.1, 0.2, 0.3, 0.4, 0.5, 0.6, 0.7, 0.8, 0.9, 1} for each variable. The selection criterion is the goodness of fit of the model characterized by the R-square. After estimating *α* for each variable, we use the formula (*Y*_*c*_ = *Y*/RR^*α*^) to adjust the time span measurement variables:

(2) PRc=PRRR0.2,Pspanc=PspanRR0.1,Tspanc=TspanRR0.5,

where Pspan and Tspan represent the lengths of P wave and T wave, respectively. Length of QRS interval is not adjusted since there is no correlation found between the QRS interval and the RR interval.

### 3.3. Sample Quantiles

**Figure 1:**
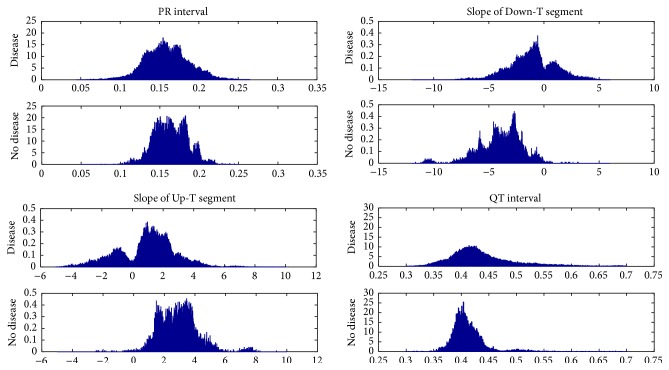
Sample distributions of the PR interval, the QT interval, the slope of the Up-T waveform, and the slope of Down-T waveform of both healthy and diseased subjects.

Each measurement variable (such as the QT interval) has one observed value per beat. Note that there are several hundred beats observed for each subject. Variation among beats can be represented by the sample distributions of the variables. To reduce the dimension and retain the key information, summary measures need to be chosen for each variable and input to a classifier. The most frequently used summary measure in ECG analysis so far is the mean of the sample distribution. However, we observe that the distributions of various measures of the diseased subjects are often skewed, more heavy-tailed, or multimodal, as compared to the symmetric, light-tailed, and unimodal distributions for healthy subjects.  Figure 1 shows the sample distributions of the PR interval, the QT interval, the slope of the Up-T waveform, and the slope of Down-T waveform of both healthy and diseased subjects. For PR and QT intervals, the distributions of the diseased subjects have heavier tails than the healthy subjects; for the slopes of Up-T and Down-T waveforms, the distributions are mixed for diseased subjects and not mixed for healthy subjects. The reason is that, for diseased subjects, most of the heartbeats are normal, with a small portion of the beats being abnormal, represented by heavy-tailed or mixed distribution for certain measurement variables. Therefore, quantiles which characterize the tail behavior of the distributions are preferred. In this paper, the 1st, 5th, 10th, 25th, 75th, 90th, and 95th percentiles, denoted by p1, p5, p10, p25, p75, p90, p95, and p99, respectively, are used to differentiate the distributions of the two groups. Further research on optimal quantile selection is ongoing.

### 3.4. Biomarker Discovery via PCA and Stepwise Discriminant Analysis

So far, six time span measurements, three amplitude measurements, and nine slope measurements are considered to be input variables for classification. For each variable, eight sample quantiles (p1, p5, p10, p25, p75, p90, p95, and p99) are used, which generates in total 144 input variables. The number of variables is relatively large compared to the number of subjects in the data set. There also exist correlations among these variables. Therefore, principal component analysis (PCA) is used to reduce the dimension and extract major information from the variables. The T wave type variable is not included in the PCA but is included in the final classification.

Table 3 displays the major quantile features in the first eight principal components. For each principal component, five variables are listed with the order determined by their coefficients. The selected features provide reasonable interpretations; for example, PC1 and PC2 mostly consist of quantiles of the QT interval and the slope of Down-T waveform. These two variables represent a significant portion of all information. To make comparisons, 10 variables selected by stepwise discriminant analysis for best classification results are listed in Table 4. The bolded variables are selected by both methods, which are more likely to be biomarkers distinguishing diseased subjects from healthy subjects. In particular, Down-T-slo_p90 plays an important role in both methods, which makes it the most significant biomarker among them.

## 4. Classification

In the classification stage, performances of four sets of input variables are compared:

- Features extracted with the mean.
- Features extracted with the mean and dimension reduced by PCA.
- Features extracted with the quantiles.
- Features extracted with the quantiles and dimension reduced by PCA.

Besides, three frequently used classifiers are compared: stepwise discriminant analysis (SDA), support vector machine (SVM), and LASSO logistic regression (LLR). As described briefly in the sequel, the three methods are based on distinctively different principles and procedures.

*(1) Stepwise Discriminant Analysis (SDA).* Discriminant analysis is a classical statistical method to separate two or more classes of objects based on the distance between them. In this paper, we develop a discriminant function using a measure of generalized squared distance. The generalized squared distance from a sample *x* to a class *t* is defined as *D*_*t*_^2^ = *d*_*t*_^2^ + *g*_1_(*t*) + *g*_2_(*t*), where *d*_*t*_^2^ is the squared Mahalanobis distance from *x* to class *t*, *g*_1_(*t*) is nonzero if the variances of different classes are unequal, and *g*_2_(*t*) is nonzero if the prior probabilities are unequal. Here *g*_2_(*t*) is ignored since equal prior probabilities are assumed. A test of homogeneity of the variances is performed to determine whether to include the item *g*_1_(*t*). To reduce the high dimensionality of the input variables, stepwise procedure is applied to select the most useful variables.

*(2) Support Vector Machine (SVM).* The idea of the support vector machines (SVMs) is to find the optimal hyperplanes between data points of different groups; see [3] for a detailed description about the method. Here the SVM classifier was implemented using LIBSVM [15], a one-against-one multiclass classifier.

*(3) LASSO Logistic Regression (LLR).* The LASSO (Least Absolute Shrinkage and Selection Operator) is a widely used shrinkage and selection method for regression models with a constraint on the sum of the absolute values of the model parameters [16]. In LASSO logistic regression, this constraint is introduced into a logistic regression model. The objective function for estimation can be expressed by adding a Lagrangian penalty to the joint log-likelihood of the model parameters [17]. In this paper, the “glmnet” package in R is used for implementing LASSO logistic regression.

**Figure 2:**
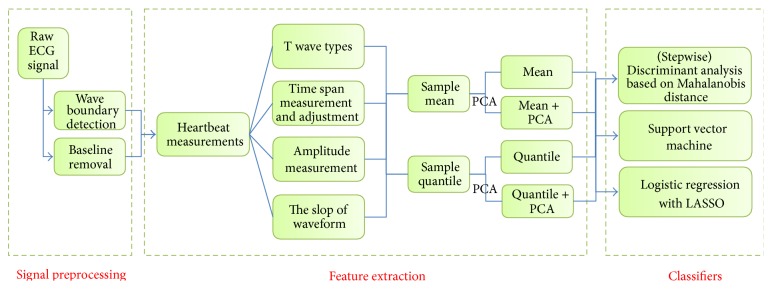
A flow chart of the classification procedure.

A summary of the procedure is shown by a flow chart in Figure 2.

## 5. Results

Results of sensitivity, specificity, and accuracy obtained on the test set of cases are displayed in Table 5. Comparing the three methods, the performance of the SDA method is better and more stable than the other two methods. The classifiers built with SVM and LLR have good sensitivity but unsatisfactory specificity. Between these two methods, results of SVM are generally better than LLR. Comparing the quantile features to the mean features, the performance of the quantile features is better and more stable than that of the mean features no matter which classifier is used. It is also found that the dimension reduction by PCA does improve the performance of all the classifiers. Therefore, the best classification procedure concluded in this paper is “Quantile + PCA + SDA” which yields 89.68% sensitivity, 84.62% specificity, and 88.52% accuracy (bolded in Table 5).

In addition to the quantile features, the T wave type variable is also useful in increasing the performance of the classifiers. In stepwise discriminant analysis, the T wave type variable is selected and increases the specificity by 7% for the quantile-based data sets and 17% for the mean-based data sets.

## 6. Discussion

Much research on ECG classification focused on beat classification; relatively little was on disease classification. To compare our results with previously reported results, we use two articles [7, 18] as examples. In [18], the authors compared the performances of logistic regression, decision trees, and neural network in disease classification and used variables not only on ECGs but also from other sources. Their sensitivity, specificity, and accuracy were all between 73.1% and 81.1%. These are lower than our results. In [7], the author developed a classification tree approach for detecting ischemia with 3-lead information on a study population of 90 subjects, the sensitivity and specificity reached 98%, higher than our results. However, since the data sets, the input variables, and the disease categories were all different, it is rather hard to compare the methods just based on results of sensitivity and specificity.

LASSO type of methods is well known for competitive performance in variable selection and classification when the number of independent variables is large (even larger than the sample size) and only a few of them are related to the response variable (model sparsity). In this application, the number of independent variables is large but still can be handled well by other methods. In addition, model sparsity may not be satisfied, because many variables may be related to the response and they are correlated. In this case, PCA and stepwise procedures are more appropriate dimension reduction methods.

Due to low sample sizes in disease categories such as bundle branch block, cardiomyopathy, and dysrhythmia, only 2-class classification is performed in the paper. However, the proposed method can be extended to multiple disease classification when more data are available. With bigger sample sizes, multilead analysis is preferred to single-lead analysis, since different diseases may show abnormality in different leads.

The performance of quantile-based measures can be improved by selecting more appropriate quantiles to distinguish the distributions of healthy and diseased subjects. Instead of using eight fixed quantiles for each variable, one may select one or two quantiles for each variable which best distinguish the distributions of that variable. Although it may take more time and effort to select optimal quantiles, both the number of variables and their correlations can be greatly reduced, extracted features will be more precise, and thus the performance of classification and biomarker identification will be greatly improved. This research is ongoing.

## Figures and Tables

**Table 1:** Number of cases in the training and testing data sets according to their class of diagnosis.

Data set	Diagnosis class	Number of cases
Training	No disease	26
	Disease	98

Testing	No disease	26
	Disease	96

**Table 2:** Definition of the nine waveforms.

Waveform	Definition
Up-P	Waveform from the start of the P wave to the peak of the P wave

Down-P	Waveform from the peak of the P wave to the end of the P wave

PR	Waveform from the end of the P wave to the start of the QRS wave

Up-R	Waveform from the start of the QRS wave to the peak of the R wave

Down-R	Waveform from the peak of the R wave to the end of the QRS wave

ST	Waveform from the end of the QRS wave to the start of the T wave

Up-T	Waveform from the start of the T wave to the peak of the T wave

Down-T	Waveform from the peak of the T wave to the end of the T wave

TP	Waveform from the end of the T wave of the current beat to the start of the P wave of the next beat

**Table 3:** Major quantile features in the first eight principal components.

Principal components	Major quantile features	Contribution (63.60%)
PC1	QT-int_p95, Down-T-slo_p95, QT-int_p90, **Down-T-slo_p90**, Down-T-slo_p75	22.62%

PC2	Down-T-slo_p25, Down-T-slo_p5, Down-T-slo_p10, Down-T-slo_p75, **Down-T-slo_p90**	10.8%

PC3	Up-R-slo_p99, QRS-amp_p99, Up-T-slo_p99, T-amp_p99, Up-T-slo_p95	9.36%

PC4	Up-P-slo_p1, P-amp_p75, PR-slo_p75, P-amp_p90, **Down-R-slo_p95**	7.12%

PC5	TP-slo_p10, TP-slo_p5, TP-slo_p25, RR-int_p90, **RR-int_p95**	5.84%

PC6	PR-int_p75, PR-int_p90, PR-int_p95, PR-int_p99, PR-int_p25	4.53%

PC7	Up-R-slo_p25, Up-R-slo_p1, P-int_p25, Down-R-slo_p25, T-amp_p99	3.32%

PC8	Up-R-slo_p1, Down-P-slo_p90, Down-P-slo_p75, Up-R-slo_p5, Down-P-slo_p95	3.00%

Note: “-int” represents the length of the indicated interval, “-slo” represents the slope of the indicated waveform, and “-amp” represents the amplitude of the indicated wave.

**Table 4:** Major features selected by stepwise discriminant analysis.

Major features	
T wave type, **Down-T-slo_p90**, Up-R-slo_p75, P-amp_p10, Up-T-slo_p90, **RR-int_p95**, QRS-amp_p1, T-int_p99, QRS-amp_p75, Down-R-slo_p99, **Down-R-slo_p95**	

Note: “-int” represents the length of the indicated interval, “-slo” represents the slope of the indicated waveform, and “-amp” represents the amplitude of the indicated wave.

**Table 5:** Classification results of the different methods on the test set of cases.

Data set	Method	Sensitivity	Specificity	Accuracy
Mean	SDA	82.29%	73.08%	80.33%
	SVM	85.57%	61.54%	80.49%
	LLR	92.71%	34.61%	80.33%

Quantile	SDA	89.58%	73.04%	86.66%
	SVM	86.6%	73.07%	83.74%
	LLR	86.46%	69.23%	82.79%

Mean + PCA	SDA	87.5%	73.08%	84.43%
	SVM	89.7%	50%	81.3%
	LLR	89.58%	38.46%	78.69%

Quantile + PCA	SDA	**89.68%**	**84.62%**	**88.52%**
	SVM	89.68%	76.92%	86.99%
	LLR	94.79%	53.85%	86.70%
